# Race, Educational Attainment, and Sustained High Body Mass Index over 24 Years of Follow-up in Middle-Aged and Older Adults

**DOI:** 10.1007/s40615-023-01589-3

**Published:** 2023-05-02

**Authors:** Shervin Assari, Sharon Cobb, Babak Najand, Hossein Zare, Amanda Sonnega

**Affiliations:** 1 Department of Urban Public Health, Charles R. Drew University of Medicine and Science, Los Angeles, CA USA; 2 Department of Family Medicine, Charles R. Drew University of Medicine and Science, Los Angeles, CA USA; 3 Marginalization-Related-Diminished Returns (MDRs) Center, Los Angeles, CA USA; 4 School of Nursing, Charles R. Drew University of Medicine and Science, Los Angeles, CA USA; 5 Department of Health Policy and Management, Johns Hopkins Bloomberg School of Public Health, Baltimore, MD 21205 USA; 6 School of Business, University of Maryland Global Campus (UMGC), Adelphi, MD 20774 USA; 7 Institute for Social Research, University of Michigan, Ann Arbor, MI USA

**Keywords:** Educational attainment, Social determinants, Race, Obesity, Middle-aged older adults

## Abstract

**Background:**

Educational attainment has been linked to reduced risk of health problems such as obesity, but research suggests that this effect may be weaker for non-Hispanic Black individuals compared to non-Hispanic White individuals, a pattern known as minorities’ diminished returns (MDRs).

**Objectives:**

This study is aimed at examining the differential association between educational attainment and sustained high body mass index (BMI) among non-Hispanic Black and non-Hispanic White middle-aged and older adults in the USA.

**Methods:**

Using data from the Health and Retirement Study (HRS) spanning 1992–2016, we analyzed a national sample of 35,110 individuals, including 7766 non-Hispanic Black and 27,344 non-Hispanic White individuals. We used logistic regression models to examine the relationship between educational attainment, race, and sustained high BMI, while controlling for age, sex, and marital status at baseline.

**Results:**

Approximately 30.6% of the sample (*n* = 10,727) had sustained high BMI, while 69.4% (*n* = 24,383) had sustained low BMI over the period of follow-up. Higher levels of educational attainment were associated with a lower risk of sustained high BMI. We also found, however, that the protective effects of education against sustained high BMI were weaker for non-Hispanic Blacks compared to non-Hispanic Whites.

**Conclusion:**

Our findings suggest that the protective effects of educational attainment against sustained high BMI may be more robust for non-Hispanic Whites than for non-Hispanic Blacks. Further research should explore whether these results are found in other racial and ethnic minorities and whether potential life history experiences may contribute to these disparities.

**Supplementary Information:**

The online version contains supplementary material available at 10.1007/s40615-023-01589-3.

## Background

Ross and Mirowsky have shown that educational attainment is among the strongest social determinants of health across various domains [1–4]. Link and Phelan’s fundamental cause [5] and Marmot’s social determinant [6, 7] frameworks also suggest that high educational attainment is associated with better physical health [8–12] as individuals who obtain higher education are more likely to have more opportunities, higher pay, and better jobs [10]. In addition to poverty [13] and economic difficulty [14], educational attainment is protective against stress [15], trauma [16], and conflict [17, 18]. A proposed biological mechanism is that high educational attainment may reduce exposure to these negative exposures and associated biomarkers [8–12]. Attaining high levels of education also reduces the risk of high body mass index (BMI) and obesity [19–22].

Yet, relatively little is known about the social determinants of sustained high BMI of middle-aged and older adults. While we know that race [23–30] and educational attainment [31–36] are associated with the odds of having high BMI, prior research is mainly focused on single observations of BMI rather than sustained high BMI over a long period of time. However, not only the level of BMI but also the duration that BMI remains high is predictive of cardiovascular mortality and morbidity [37]. In addition, while Fryar et al. and Ogden et al. have shown social inequalities in BMI by race and education [38–42], most previous studies on this topic have examined the separate or additive effects of race and educational attainment on BMI. Although we know that non-Hispanic Blacks are more likely to have high BMI compared to non-Hispanic White individuals [23–30], and high educational attainment is associated with lower BMI [31–36], an important remaining question is whether race moderates the association between educational attainment and sustained high BMI over a long time period.

A recent body of research has shown that higher education shows stronger associations with health for non-Hispanic Whites than for non-Hispanic Blacks, a pattern called minorities’ diminished returns (MDRs) [43]. Minorities’ diminished returns is a term used to describe the observation that even when minority individuals achieve the same level of education and income as non-minority individuals, they still face systemic obstacles and barriers that limit their social and economic mobility. These obstacles may include discrimination, racial bias, cultural barriers, and lack of access to social networks and resources. The MDR phenomenon refers to lower socio-economic and health outcomes that members of racial minority groups often experience despite achieving similar levels of education or income as non-minority individuals. MDRs can manifest in several ways, such as lower rates of homeownership, higher levels of poverty, and lower levels of upward mobility within the job market of highly educated Black individuals compared to highly educated Whites. This phenomenon is a reflection of structural racism and systemic inequities that perpetuate racial disparities across class lines [43].

Research investigating the impact of MDRs demonstrates that educational attainment has stronger positive effects on BMI [43], diet [44], and exercise [45] for non-Hispanic Whites than for non-Hispanic Blacks. Thus, it is plausible to expect diminished returns of educational attainment on sustained high BMI for non-Hispanic Blacks relative to non-Hispanic Whites. Given the relative paucity of studies on sustained high BMI, in general, and the importance of further elucidation of MDRs, this study fills a significant gap: to study the nature of racial differences in the association between educational attainment and sustained high BMI in middle-aged and older adults.

Knowledge regarding racial variation in the protective effects of SES indicators such as educational attainment against health problems such as high BMI will have public health implications because, historically, equalizing SES is assumed to be the solution for racial health disparities [43, 46, 47]. Knowledge about racial variation in SES effects on health could potentially improve research and public health practices and policy making to address structural barriers that may impact racial minorities across all SES levels [43, 46, 47]. In addition, documenting that it is not merely SES that contributes to racial health inequality may provide evidence that racial health disparities may be due to the legacy of racism and slavery in the lives of non-Hispanic Black Americans. Our research contributes to a growing understanding that policies aimed at reducing racial gaps in SES may not be sufficient to close health gaps. It also embraces the growing understanding that little progress will come from merely continuing to include race as a covariate in racial inequality research.

### Aim

The aim of this study is to test the variation between non-Hispanic White and non-Hispanic Black and middle-aged and older adults in the protective effects of educational attainment on the odds of having sustained high BMI.

## Methods

### Design and Setting

Data came from the Health and Retirement Study (HRS) [48–57], 1992 to 2016. HRS has conducted biennial interviews of a nationally representative sample of US adults over the age of 50. The original HRS cohort was born 1931 to 1941, and new cohorts have been added every 6 years since 1998. The study includes a wide range of content relevant to aging, collected in a multi-modal context. Researchers at the RAND Center for the Study of Aging create a user-friendly version of much of the core data, known as the RAND HRS. Although more information is available elsewhere [53], we provide a summary of the key features of the study here. The HRS is sponsored by the National Institute on Aging (grant number NIA U01AG009740) and is conducted by the University of Michigan.

### Participants and Sampling

This study included all available cohorts through the late baby boomers, recruited in 2016. The HRS sampling strategy was a national area probability sample of all US households. Using this national sampling frame, interviewers identified an age-eligible participant (51–56 years for each 6-year birth cohort) in the household. If the sampled respondent has a spouse or partner, that individual is invited to participate, regardless of their age.

### Analytical Sample

The current study included individuals who were 51+ years old (at their baseline interview) and self-identified as White or Black. Given the focus of this investigation as being race rather than ethnicity, only non-Hispanic individuals were included in our analysis. This sample included all participants who had at least one BMI data point. We also excluded participants below the age of 51. As described below, we used latent class analysis which allowed us to include all participants regardless of the duration of follow-up. The average number of waves of follow-up was 6(SD = 4). This method significantly reduces attrition bias over the long period of follow-up. Less than 5% of our original sample who were eligible for our analysis were dropped from the analysis due to missing data. Individuals who were not included in our analysis were more likely to be non-Hispanic Black and low educated.

### Data Collection

The HRS features rich measurement across a wide range of domains. For the purposes of the present study, we utilized data from the following domains: demographic, SES, and health information (BMI). The data used in this study were collected through either telephone or in-person interviews. We used the RAND HRS Longitudinal File [58], which included all the variables needed for this study.

### Measures

#### Race

Race was measured at baseline with a question that allowed respondents to indicate whether they considered themselves to be White/Caucasian, Black/African American, American Indian or Alaskan Native, Asian or Pacific Islander, or other. Another question allowed respondents to indicate whether they identified as Hispanic or Latino.

Educational attainment was assessed in the survey as the number of years of education. Education categories were as follows: lowest (some high school), low (graduated high school), high (completed some college), and highest education (graduated college). We did not define an SES index because correlations between education and income/wealth are racialized, so an index such as this would not be useful in comparing across racial categories [59].

#### Body Mass Index

Participants in the HRS self-reported their height and weight at each biennial interview from 1992 to 2016 (W1–W13). BMI was calculated based on the conventional definition (weight (kg)/[height (m)]^2)^. We used a cluster analysis to define a dichotomous variable based on BMI measures over time. These class memberships reflected sustained high and sustained low BMI, with an average range from 32.37 kg/(m)]^2^ to 35.01 kg/(m)]^2^ and 24.60 kg/(m)]^2^–25.67 kg/(m)]^2^, respectively [60–62]. We coded those in the sustained high class membership as 1 and those in the sustained low class membership as 0.

### Data Analysis

For continuous variables such as age and BMI, we reported mean and standard deviation (SD). For categorical variables such as race, education, and marital status, we reported the frequency and relative frequency (%). All descriptive data were reported overall and then by race. We used chi-square tests to compare study variables between non-Hispanic Whites and non-Hispanic Blacks. For multi-variable modeling, we ran logistic regression models. The main predictor variable was educational attainment. The moderator was race. The outcome variable was sustained high BMI, treated as a binary variable, reflecting the class membership in latent class analysis. Covariates included sex, age, and baseline marital status. We compared non-Hispanic Blacks and non-Hispanic Whites for the effects of educational attainment on odds of sustained high BMI. To avoid over-adjustment, we did not control for other mechanisms such as diet, exercise, or food environment [63, 64]. Those differ based on race and educational attainment, but their differences may be the reason why Black-White differences exist in the health returns of education.

The first logistic regression models were fitted to the pooled sample in the absence and presence of SES indicators by race interaction terms. The first model included race and educational attainment. In Model 2, we added an interaction term that was the multiplicative product of race (= 1, non-Hispanic Blacks, = 0, non-Hispanic Whites) and educational attainment. Because the interaction between race and education was significant (*p* < 0.001), we stratified the model by race (Model 3 and Model 4). In all models, we controlled for sex, age, and marital status. Before we estimated our models, we ruled out multi-collinearity between race, educational attainment, and BMI. After pooled sample models, we also tested logistic regressions that were stratified by race. A *p*-value of less than or equal to 0.05 was statistically significant. We reported odds ratio (OR), standard errors (SE), confidence intervals (CI), and *p*-values.

## Ethics Statement

The HRS study protocol was approved by the University of Michigan (UM) Institutional Review Board (IRB). All HRS participants signed a written consent. The data were collected, stored, managed, and analyzed in a fully anonymous fashion. We did not need to have additional ethics approval because we used the publicly available data.

## Results

### Descriptive Data Overall

Overall, 35,110 White or Black middle-aged and older adults were included in our analysis. Seven thousand seven hundred sixty-six Black and 27,344 White individuals were included. From this number, 30.6% (*n* = 10,727) had consistently high and 69.4% (*n* = 24,383) had sustained low BMI. This study included between 11,189 and 19,056 individuals per wave. 27.2% (*n* = 7424) of Whites and 42.5% (*n* = 3,303) of Blacks had sustained high BMI. In the full sample, about 32% had sustained high BMI. Table 1 shows the average BMI of participants at each wave Table 2.

**Table 1:** Number of participants and mean (SD) of body mass index (BMI) over time

	*n*	Mean	SD
1992	11,189	27.1	5.2
1994	17,617	26.4	5.0
1996	16,092	26.5	5.1
1998	19,056	26.8	5.2
2000	17,372	27.0	5.4
2002	16,021	27.1	5.5
2004	17,409	27.4	5.7
2006	16,068	27.8	5.9
2008	14,958	27.9	5.9
2010	18,106	28.4	6.2
2012	16,847	28.4	6.2
2014	15,254	28.5	6.3
2016	16,200	28.9	6.4

**Table 2:** Average body mass index (BMI) of low sustained BMI and high sustained BMI groups overall and by race and education

	All	Low BMI	High BMI	Lowest education		Low education		Mid education		High education		Highest education		Non-Hispanic White		Non-Hispanic Black	
				Low BMI	High BMI	Low BMI	High BMI	Low BMI	High BMI	Low BMI	High BMI	Low BMI	High BMI	Low BMI	High BMI	Low BMI	High BMI
1992	27.1	24.6	32.4	24.8	33.2	24.7	32.2	24.6	32.2	24.5	31.9	24.5	31.9	24.5	32.0	25.2	33.5
1994	26.4	24.4	32.6	24.4	32.9	24.7	32.4	24.4	32.6	24.3	32.3	24.3	31.9	24.3	32.3	25.1	33.4
1996	26.5	24.4	32.8	24.3	33.3	24.8	32.4	24.4	32.8	24.3	32.5	24.3	32.2	24.3	32.5	25.0	33.6
1998	26.8	24.5	33.0	24.4	33.4	25.0	32.8	24.5	33.0	24.5	32.8	24.5	32.7	24.4	32.8	25.0	33.7
2000	27.0	24.6	33.3	24.6	33.7	25.2	33.1	24.6	33.4	24.6	33.3	24.5	33.0	24.5	33.2	25.2	34.0
2002	27.1	24.7	33.5	24.6	33.8	25.1	33.4	24.7	33.6	24.7	33.6	24.6	33.0	24.6	33.3	25.2	34.2
2004	27.4	24.7	33.8	24.6	34.4	25.1	33.6	24.7	33.8	24.7	33.9	24.6	33.1	24.6	33.6	25.3	34.6
2006	27.8	25.0	34.3	24.9	34.6	25.2	34.3	25.0	34.2	25.1	34.5	24.9	33.8	24.9	34.1	25.4	34.9
2008	27.9	25.1	34.4	24.9	34.5	25.4	34.2	25.0	34.4	25.2	34.6	25.0	34.1	25.0	34.2	25.4	34.9
2010	28.4	25.1	34.8	24.9	34.8	25.4	35.0	25.1	34.8	25.3	34.9	25.1	34.5	25.1	34.5	25.5	35.6
2012	28.4	25.1	34.8	24.9	34.5	25.5	34.8	25.1	34.8	25.3	35.0	25.0	34.5	25.0	34.5	25.5	35.3
2014	28.5	25.2	34.8	25.1	34.3	25.4	34.9	25.2	35.0	25.3	35.0	25.0	34.6	25.1	34.5	25.5	35.3
2016	28.9	25.3	35.0	25.0	34.5	25.4	35.4	25.3	35.0	25.4	35.3	25.2	34.8	25.2	34.7	25.6	35.6

We used a cluster analysis to define a dichotomous variable based on BMI measures over time, reflecting sustained high and sustained low BMI. Lowest education corresponds to some high school; low education corresponds to high school graduation; high education corresponds to some college; and highest education corresponds to college graduation

Supplementary figure 1 shows average BMI overall. Supplementary figure 2 shows average BMI of our BMI membership groups. As Supplementary figure 3 shows, education groups clustered together within sustained low and sustained high BMI.

### Pooled Sample Models

In the pooled sample without interactions, educational attainment was inversely associated with sustained high BMI (Table 3, Model 1, main effects). Specifically, compared to those with less than high school educational attainment, those with some college and college graduates had lower odds of having sustained high BMI (Model 1).

**Table 3:** Association between educational attainment and sustained high BMI in American middle-aged and older adults by race

	Model 1				Model 2			
	Main effects				Interactions			
	Odds ratio	95% CI		*p*	Odds ratio	95% CI		*p*
Non-Hispanic Black (Ref. non-Hispanic White)	1.63	1.47	1.81	0.000	1.52	1.28	1.79	0.000
Age	0.97	0.97	0.98	0.000	0.97	0.97	0.98	0.000
Male (Ref. female)	0.98	0.90	1.07	0.696	0.99	0.91	1.08	0.819
No education (Ref.)	--	--	--	--	--	--	--	--
Some high school	1.17	0.97	1.42	0.096	1.14	0.92	1.40	0.229
Graduated high school	0.92	0.82	1.02	0.111	0.89	0.79	1.02	0.083
Some college	0.80	0.71	0.91	0.001	0.79	0.69	0.92	0.002
Graduated college	0.65	0.57	0.74	0.000	0.60	0.51	0.7	0.000
Interaction race and education	--	--	--	--	--	--	--	--
NH Black with some high school	--	--	--	--	1.10	0.68	1.78	0.712
NH Black graduated high school	--	--	--	--	1.06	0.82	1.35	0.674
NH Black with some college	--	--	--	--	0.99	0.73	1.33	0.919
NH Black graduated college	--	--	--	--	1.66	1.13	2.34	0.004

There was a statistically significant interaction between race and the highest educational attainment (college graduate) (Table 3, Model 2, interactions). This interaction was suggestive of higher odds of sustained high BMI in non-Hispanic Blacks with a college education compared to non-Hispanic Whites with similar education (Table 3).

### Race-Specific Models

Given that the interaction between education and race was statistically significant (*p* = 0.004), we stratified the analyses by race. We found that highest level of educational attainment (college graduation) was associated with lower sustained high BMI for non-Hispanic Whites but not for non-Hispanic Blacks (Table 4).

**Table 4:** Association between educational attainment and sustained high body mass index (BMI) in American middle-aged and older adults by race

	Model 3				Model 4			
	Non-Hispanic White				Non-Hispanic Black			
	Odds ratio	95% CI		*p*	Odds ratio	95% CI		*p*
Age	0.97	0.96	0.98	0.000	0.98	0.96	0.99	0.004
Male (ref. female)	1.17	1.06	1.29	0.001	0.53	0.44	0.64	0.000
Education								
No education (Ref.)	--	--	--	--	--	--	--	--
Some high school	1.12	0.91	1.39	0.280	1.19	0.76	1.85	0.451
Graduated high school	0.90	0.80	1.03	0.118	0.90	0.72	1.12	0.341
Some college	0.79	0.69	0.92	0.002	0.75	0.58	0.98	0.037
Graduated college	0.59	0.51	0.68	0.000	0.91	0.67	1.25	0.575

## Discussion

In this study, we aimed to investigate the joint effects of race and educational attainment on sustained high BMI over a long period of time in US middle-aged and older adults. We found that (1) non-Hispanic Black race is associated with higher BMI net of education and (2) the returns of education in terms of reducing odds of sustained high BMI is weaker for non-Hispanic Black than non-Hispanic White individuals.

Education is considered among the strongest and most robust protective factors for health outcomes as individuals who have higher educational attainment have more opportunities in life, work in better jobs, have better access to health care services, show better health behaviors such as diet and exercise, have less stress, and live in better neighborhoods [1–10, 21–23]. We explored racial variations in the effects of education on high sustained BMI in US middle-aged and older adults. In a national sample of American adults, race and educational attainment showed interdependent (interactive) in addition to additive effects on the level of sustained BMI. The protective effects of education were weaker for non-Hispanic Blacks than non-Hispanic Whites.

Our observation that educational attainment has more salient protective and preventive roles for maintaining a healthy BMI for non-Hispanic Whites than non-Hispanic Blacks is in line with the MDRs framework [65], suggesting that individual-level protective social determinants such as educational attainment have systematically weaker health effects for non-Hispanic Blacks than for non-Hispanic Whites individuals.

MDR theory suggests that racial variation in the returns of SES emerges because of racism, segregation, and social stratification. Extensive work on the MDR phenomenon has shown worse-than-expected health of middle-class non-Hispanic Blacks across studies, age groups, outcomes, and settings [66, 67]. As a result of MDRs, the same change in SES results in a smaller change in health outcomes in non-Hispanic Blacks than in non-Hispanic Whites. Racism, stratification, and segregation are all argued to limit the return of education for Black communities [68].

According to the MDR theory, several SES resources lose some of their strength in the presence of structural racism [43]. Structural racism refers to the totality of ways in which US society has fostered and is continuing to foster racial discrimination through mutually reinforcing systems of housing, education, employment, earnings, and policing. Due to structural racism, non-Hispanic Black people continue to experience high levels of obstacles, regardless of their education [69, 70]. For non-Hispanic White families, however, higher educational attainment may lead to better job opportunities, better living neighborhoods, better social environment, and lower stress [65]. However, the health status and health needs of non-Hispanic Blacks and non-Hispanic Whites with the same SES highly differ [71]. As a result, needs for public health programs are not low for middle-class non-Hispanic Black communities. This is an important implication of this work and has relevance for research, practice, and program planning [72]. Research-based knowledge that informs us on how middle- and high- SES non-Hispanic Blacks and non-Hispanic Whites differ in the risk of obesity and cardiovascular conditions has implications for the prevention, detection, diagnosis, and treatment of cardiovascular and metabolic diseases. High BMI is known to have a role in physiopathology of chronic diseases such as cancer, diabetes, hypertension, heart disease, and stroke that are all disproportionately more common for racial minorities such as Blacks.

Structural racism [73] creates an environment that increases disparities. Structural racism results in segregation, which is in turn linked to gaps in employment [74, 75], income [76], and wage [77] disparities between Black and White people. Long-standing structural racism has created deeply rooted Black-White social and economic disparities that result in Black-White health inequities [74]. Some elements of structural racism are that Black individuals work in worse jobs than Whites [78], and Black individuals earn less income than Whites [79], which directly and indirectly influence racial differences in health and illness [80].

## Implications

As our findings suggest, likely due to structural racism, high educational attainment (college graduation) may be more beneficial to non-Hispanic Whites’ than non-Hispanic Blacks’ BMI over a long period of time. It is essential to tailor our diagnostic and preventive programs and health services for racial groups who are even middle class, simply because racial gaps in health are not limited to those who are poor. Racial differences exist in middle- and high-SES groups; thus, they also need our policy attention and program planning [72]. This study demonstrated the differential protective effects of high educational attainment against the odds of having high sustained BMI among non-Hispanic Blacks and non-Hispanic Whites. Studies have shown differences between non-Hispanic Whites and non-Hispanic Blacks in risk and protective factors that correlate with obesity [81]. Thus, researchers, clinicians, and practitioners should be aware that one size does not fit all, and these heterogeneities require policies that address the needs of middle-class non-Hispanic Black people.

## Limitations

The present study had a few methodological limitations and weaknesses. First, this study was limited because of its observational design, which means we are unable to draw causal inferences between educational attainments and BMI. The second limiting factor was the lack of covariates at the neighborhood level and also the lack of measures such as diet, food norms, and available food outlets in the residential area. Environmental factors such as neighborhood poverty or proximity to healthy grocery stores and greens and parks may have implications for BMI. However, we carefully limited our covariates to avoid over-adjustment [82]. Another strength was a large diverse sample size and longitudinal design.

## Future Research Directions

This analysis was limited to individual-level data. Non-Hispanic Black and non-Hispanic Whites also differ in proximity to fast food stores and their neighborhood walkability. There is also a need to conduct studies on changes in BMI over time. There is a need for future studies on other racial and ethnic minorities, especially Hispanic individuals. Future research should also consider within-race heterogeneity in non-Hispanic Black Americans. For example, experiences and exposures of US-born and immigrant non-Hispanic Black Americans are widely different.

## Conclusion

While highly educated non-Hispanic White middle-aged and older adults are protected against risk of high sustained BMI, highly educated non-Hispanic Blacks remain at risk of high BMI over a long period of time. This paradox, also known as minorities’ diminished returns, may reflect structural racism and social stratification in the USA.

### Supplementary Information

Below is the link to the electronic supplementary material.

Supplementary file1 (DOCX 31 KB)

## Data Availability

Data available at https://wwwn.cdc.gov/nchs/nhanes/.
