# Supplementary material for: Race, Educational Attainment, and Sustained High Body Mass Index over 24 Years of Follow-up in Middle-Aged and Older Adults
Source: J Racial Ethn Health Disparities. 2023 May 2;11(2):1097–105. doi: 10.1007/s40615-023-01589-3 (PMC10933141; doi:10.1007/s40615-023-01589-3)
Supplement: Supplementary file 1 — Supplementary file1 (DOCX 31 KB) [file 40615_2023_1589_MOESM1_ESM.docx]

Supplementary Figures

**1-a) BMI trajectory overall**

1-b) BMI trajectory across low and high sustained BMI groups

**Figure 1.** Average BMI overall (1-a, blue) and average BMI of high sustained BMI (1-b, orange) and average BMI of sustained low BMI (1-b, blue) (x = wave number, y = average BMI)

**Figure 2.** Whites with high sustained BMI (gray), Whites with low sustained BMI (blue), Blacks with high sustained BMI, Blacks with low sustained BMI (x = wave number, y = average BMI)

**Figure 3.** Individuals with high sustained BMI across educational attainment (red, orange, and yellow shades) and individuals with low sustained BMI (navy, blue, and gray shades) across education levels (x = wave number, y = average BMI)
